# The association between the child’s age and mothers’ physical activity: results from the population-based German National Cohort study

**DOI:** 10.1186/s12889-024-19055-y

**Published:** 2024-06-13

**Authors:** Lisa Scharfenberg, Sarah Negash, Alexander Kluttig, Rafael Mikolajczyk

**Affiliations:** Institute of Medical Epidemiology, Biometry and Informatics, Martin-Luther-University Halle-Wittenberg, Magdeburger Str. 8, 06112 Halle (Saale), Germany

**Keywords:** Motherhood, Physical activity, Leisure-time, Transport, Work

## Abstract

**Background:**

Since physical activity is an important determinant of physical and mental health, lower levels of physical activity among mothers reported in previous research are concerning. The aim of this study was to examine whether physical activity levels differ among mothers depending on the age of the youngest child.

**Methods:**

Cross-sectional data from the German National Cohort study, comprising 3959 mothers aged 22–72 years with offspring aged 0–54 years (grouped into 0–5, 6–11, 12–17, 18–29 and > 30 years) was used. The Global Physical Activity Questionnaire (GPAQ) was used to assess physical activity among mothers in leisure time, transport and (occupational and non-occupational) work settings, quantified as MET-minutes per week. Means (with 95% confidence interval) of mothers’ weekly MET-minutes were visualized in graphs, stratified by mothers’ and the youngest child’s age. Linear regression analyses assessed the association between the child’s age and self-reported time and intensity of mothers’ physical activity within each activity domain and for the total physical activity.

**Results:**

Adjusted results suggested that the MET-minutes in work settings were lower among mothers with younger children. This association was clearest in mothers whose youngest child was under 12 years old, among whom lower self-reported physical activity at work compared to mothers with children at age 30 and older was found. No association was observed between the age of the youngest child and mothers’ MET-minutes in leisure nor in transport settings. The self-reported physical activity of mothers whose youngest child was in the same child age group was found to be lower with increased maternal age. As expected, the work related activity dominated the self-reported physical activity.

**Conclusions:**

The results show differences in mothers’ self-reported physical activity by the age of the youngest child. The strongest difference was related to physical activity in work settings, indicating the need for supportive actions.

**Supplementary Information:**

The online version contains supplementary material available at 10.1186/s12889-024-19055-y.

## Background

Physical activity is one of the key modifiable health risk factors for non-communicable diseases [1–5]. Not only its importance for longevity [5], but also for mental health [6, 7] and well-being [8] is well-established. Therefore, attention must be paid to the lower general physical activity levels in parents when compared to their childless counterparts [9, 10]. Particularly mothers fail to meet the WHO recommendations for physical activity of at least 150 min moderate or 75 min vigorous physical activity per week [2, 11]. Furthermore, the relationship between motherhood and physical activity seem to differ by the age of the youngest child [11–14]. While some research reported a stronger association between being a mother and physical activity among mothers with younger children [11–13], only one study investigated the direct relation between mothers’ physical activity and the child’s age, while differentiating between school-aged and younger children [14]. Overall, physical activity levels were found to be lowest among mothers with a child under 6 [11–13] or, respectively, under 5 years old when compared to mothers of older children [14]. However, how mothers’ participation in physical activity changes when their offspring grow older remains unclear. Kendig et al. investigated differences in physical activity levels among women aged 65 years and older with and without children [15]. Contrary to the findings in younger mothers, physical activity in later life was higher among mothers than among childless women [15]. The age of the child(ren) was not considered in this study, however, since the mothers were aged 65 and older, the majority of their children may have reached adulthood. Collectively, these studies outline the critical role of the child’s age for mothers’ participation in physical activity. Still, the association of the child’s age with mothers’ physical activity is understudied, even though it is known that adult offspring affect parents’ lives in other ways than minor children [15, 16].

WHO defined three main types of physical activity: leisure time, transport-related, and work-related [17]. These physical activity domains are potentially affected by having children in different ways [18]. Most studies on physical activity in relation to child`s age did not distinguish different domains of physical activity [12–14, 19–21]. The majority of research has focused on active leisure among mothers, showing a clear association between being a mother and lower physical activity levels in this domain [11, 22, 23], whereas studies on physical activity in transport and work settings among mothers were inconclusive [18, 24]. A systematic understanding of the relationship between physical activity domains and motherhood is still lacking.

Our aim is to investigate how mothers’ physical activity levels differ with the age of the youngest child. We advance the existing literature by taking mothers of offspring aged between 0 and 54 years into account and examining leisure time, transport-related and work-related physical activity separately.

## Methods

### Study population

We used data from the baseline examination from the study center in Halle (Saale) of the German National Cohort (GNC), a population based cohort study that was set out to gain deeper insight into causes of major chronic diseases. More detailed information about the study was published elsewhere [25, 26]. The baseline assessment took place between 2014 and 2019. All participants underwent a standardized computer-assisted personal interview, self-administrated questionnaires and standardized physical examinations. The study population included in this paper comprises 5251 women, of whom 4041 were mothers (experienced at least one live birth) and 3959 provided information on the time of birth of their children. Hence, 3959 women between the ages of 22 and 72 were included in our analyses. Informed consent was obtained from all subjects involved in the GNC. The study was conducted according to the guidelines of the Declaration of Helsinki, and approved by the Ethics Committee of the Martin Luther Universität Halle-Wittenberg (Halle (Saale), Germany). An “ethics code”, covering general principles and rules for ethical assessment and handling of study data, was developed for the study [27].

### Age of the youngest child

To assess the age of the youngest child, we subtracted the age at their last (life) birth from the mothers’ age. Mothers were asked to indicate the age at their last birth as integer value. The resulting inaccuracy was corrected by adding 0.5 years to the age of the youngest child. We classified the child’s age into five groups: 0–5, 6–11, 12–17, 18–29, and ≥ 30 years. Trough all the subsequent analyses, the age of the youngest child was used as categorical variable.

### Physical activity

Physical activity was assessed using the standardized Global Physical Activity Questionnaire (GPAQ) [28, 29]. It provides information on self-reported weekly time and intensity spent in three domains of physical activity: physical activity in leisure time, for transport (travel to and from places) and at work. The variable work-related activity includes, besides occupational physical activity, unpaid work activities such as household chores or harvesting food [28]. According to the WHO GPAQ Analysis Guide [28], after cleaning the data for missing and out-of-range values, the metabolic equivalent (MET)-minutes per week were computed within each category of physical activity. The MET value 4 was used for moderate physical activity in leisure and work settings and for transport-related physical activity. The MET value 8 was used for leisure-time and work-related vigorous physical activity. A person who bicycles 60 min per week to get to and from places achieved 240 MET-minutes in the category transport-related physical activity. If participants indicated not to be active in one domain, the MET value amounted to 0 MET-minutes. To address the issue that a considerable proportion of participants indicated not to perform any activity in the specific domain, we generated binary variables for being active at the particular domain of physical activity (yes vs. no). In the questionnaire, to be physically active was defined as at least 10 min continuous activity that causes large increases in respiratory or heart rate [28]. For the subset of participants who indicated to be active in the particular domain, we quantified the weekly energy expenditure for moderate to vigorous physical activity. These variables were used to characterize leisure time, transport- and work-related physical activity in the sensitivity analyses. By adding up the MET values of the three activity categories, we obtained information on mother’s total weekly physical activity.

### Other variables

In the analyses, we included mothers’ age, the partner status, years of education and the self-rated health status as covariates. We selected these variables based on a comprehensive literature review (e.g. [30–33]). The mothers’ age in years was computed by subtracting the birth date from the examination date. We further generated an ordinal variable with 10-year age groups for the mothers’ age, whereby the ages between 60 and 72 were cumulated. The grouped variable for mother’s age was used in the analyses. To consider the potentially confounding effect of having a partner, we generated a variable for partner status with three categories according to the guidelines for assessment of sociodemographic characteristics in the GNC [34]. Participants with partners were grouped into “living with a partner” and “not living with a partner”. In doing so, irregular cohabitation (e.g. only on weekends) was categorized into “not living with a partner”. The third category comprised participants without partner. The highest level of school and vocational education was defined according to the International Standard Classification of Education (ICSED, 1997) [34, 35]. Accordingly, we computed the years of education related to each educational level. For instance, the highest level was “doctoral degree” and related to 20 educational years. The eight participants without any finished school or vocational education were not included in our analyses. For information on the self-rated health status, participants were asked to rate their general health. The five response options were dichotomized into “good” (fair, good, very good) and “poor” (poor, very poor), in compliance with other studies [36, 37] and the GNC standards [34].

### Statistical analysis

Frequencies and means with standard deviations were used to describe the characteristics of the sample. We estimated the means for MET-minutes in work, transport, leisure time and total physical activity stratified by the grouped mother’s age and the grouped child’s age, to show possible effects of the child’s age on mothers’ physical activity levels independent of mothers’ age. The stratified mean MET-minutes with belonging 95% confidence intervals were visualized in graphs. Due to small sample size in some categories of child’s and mothers’ age, the mean MET-minutes are reported for groups of 30 participants at minimum. Linear regression models were used to study the association between the age of the youngest child and the weekly MET-minutes within the three physical activity categories and for total physical activity. The linear regression analyses were performed for mothers between the ages 30 and 59, due to small variation in child’s age among younger and older mothers. Due to asymmetrical distribution of the MET-minutes variables, we performed sensitivity analyses by excluding the participants who indicated not to have any physical activity in the particular domain. Firstly, all regression analyses were performed unadjusted. Then, we adjusted for mothers’ age, the partner status, educational years, and the self-rated health status. All analyses were performed using IBM SPSS Statistics 28 [38].

## Results

### Descriptive statistics

As shown in Table 1, the mothers were between 22 and 72 years old (mean: 53.0 with a standard deviation (SD): 10.2). The age of the youngest child ranged from 0 to 54 years with mean age of 25.8 (SD: 12.6) years. More than two thirds of the mothers’ youngest child aged 18 and older. Nearly half of the mothers had two children, 39.4% one child and 12.5% three or more children. As for demographic factors, 75.2% were living with a partner, the mean education years amounted to 15.5 (SD: 2.0) and 11.5% of the mothers indicated to have poor health. Overall, 93.8% of the mothers were active in at least one of the three domains (Table 2). The proportion to be active at the particular domain of physical activity was 48.6% for work-related physical activity, 67.6% for transport-related physical activity and 74.0% for leisure time physical activity. With respect to all participants, the mean MET-minutes per week were 1662 (SD: 3069) minutes per week spent in leisure time physical activity, 1702 (SD: 2896) in transport-related physical activity, 4375 (SD: 7832) in work-related physical activity and 7716 (SD: 9857) total MET-minutes per week. Among the participants who indicated to have any activity, the mean MET-minutes per week amounted to 2259 (SD: 3386) minutes per week spent in leisure time physical activity, 2536 (SD: 3227) in transport-related physical activity, 9140 (SD: 9220) in work-related physical activity and 8259 (SD: 9975) total MET-minutes per week.

**Table 1:** Characteristics of the sample (*N* = 3959)

Variables	N	% or Mean (SD)	Missing %
Mother’s age (years)			0
22–29	75	1.9%	
30–39	324	8.2%	
40–49	1143	28.9%	
50–59	1264	31.9%	
60–72	1153	29.1%	
Mean	3959	53.1 (10.2)	
Partner status			0.4
Partner, living together	2962	75.2%	
Partner, not living together	315	8.0%	
No partner	649	16.5%	
Years of education (isced97)	3722	15.5 (2.0)	6.0
Poor self-rated health	450	11.5%	0.5
Age of the youngest child			0
0–5	329	8.3%	
6–11	380	9.6%	
12–17	482	12.2%	
18–29	1074	27.1%	
≥ 30	1694	42.8%	
Mean	3959	25.8 (12.6)	
Number of live births			0
1	1554	39.4%	
2	1894	48.1%	
3 +	493	12.5%	

*SD* Standard deviation

**Table 2:** Descriptive statistics of the physical activity variables (*N* = 3959)

Physical activity	N	% or Mean (SD)	Missing %
Leisure time (MET-minutes/week)	3848	1662 (3069)	2.8
Active in leisure time	2914	74.1%	
MET-minutes > 40 MET^a^	2814	2259 (3386)	
Transport (MET-minutes/week)	3884	1702 (2896)	1.9
Active in transport	2659	67.6%	
MET-minutes > 40 MET^a^	2594	2536 (3227)	
Work (including housework) (MET-minutes/week)	3886	4375 (7832)	1.8
Active at work	1911	48.6%	
MET-minutes > 40 MET^a^	1852	9140 (9220)	
Total (MET-minutes/week)	3747	7716 (9857)	5.4
Active at any PA domain	3704	93,8%	
MET-minutes > 40 MET^a^	3501	8259 (9975)	

*MET* Metabolic equivalent

*SD* Standard deviation

^a^calculated only for those who indicated to be active at the particular mode of physical activity (> 40 MET-minutes)

### The association between child’s age and maternal physical activity

**Fig. 1:**
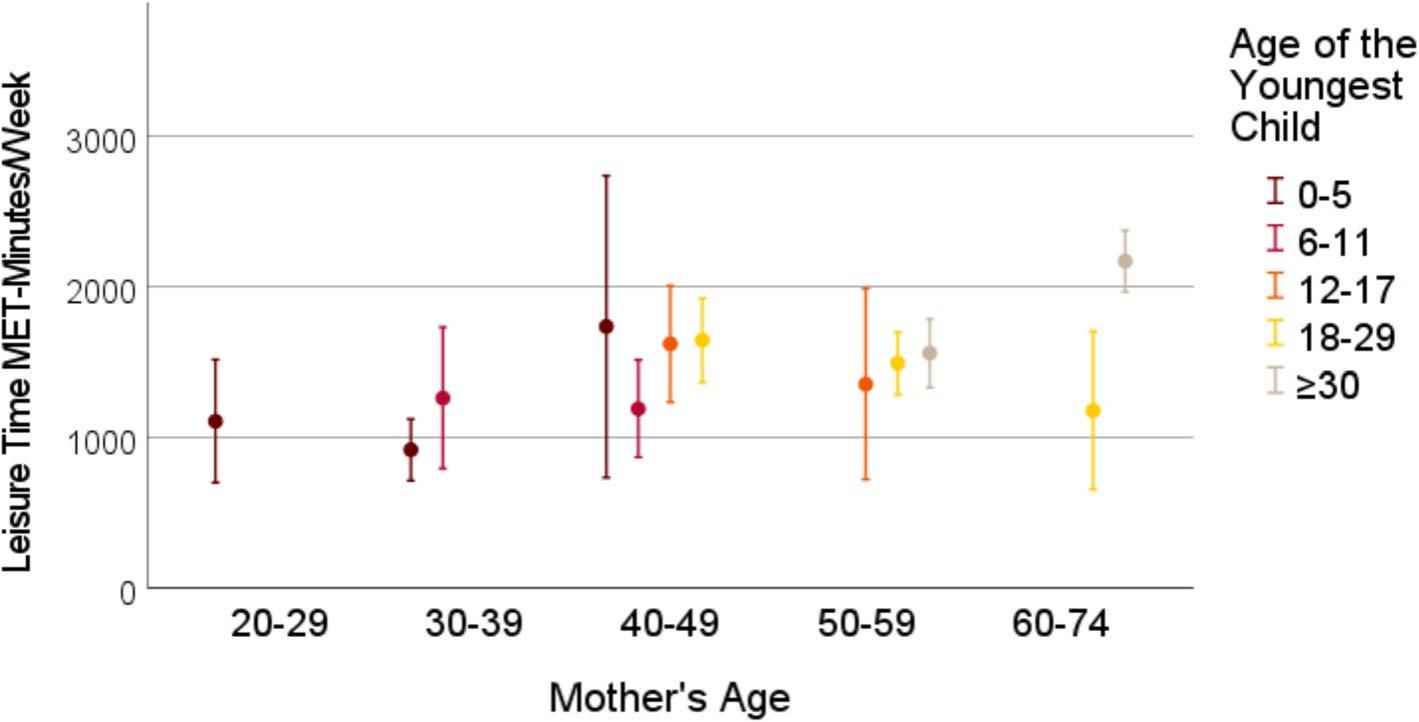
Leisure time MET-minutes per week for subgroups with N > 30 mothers

**Fig. 2:**
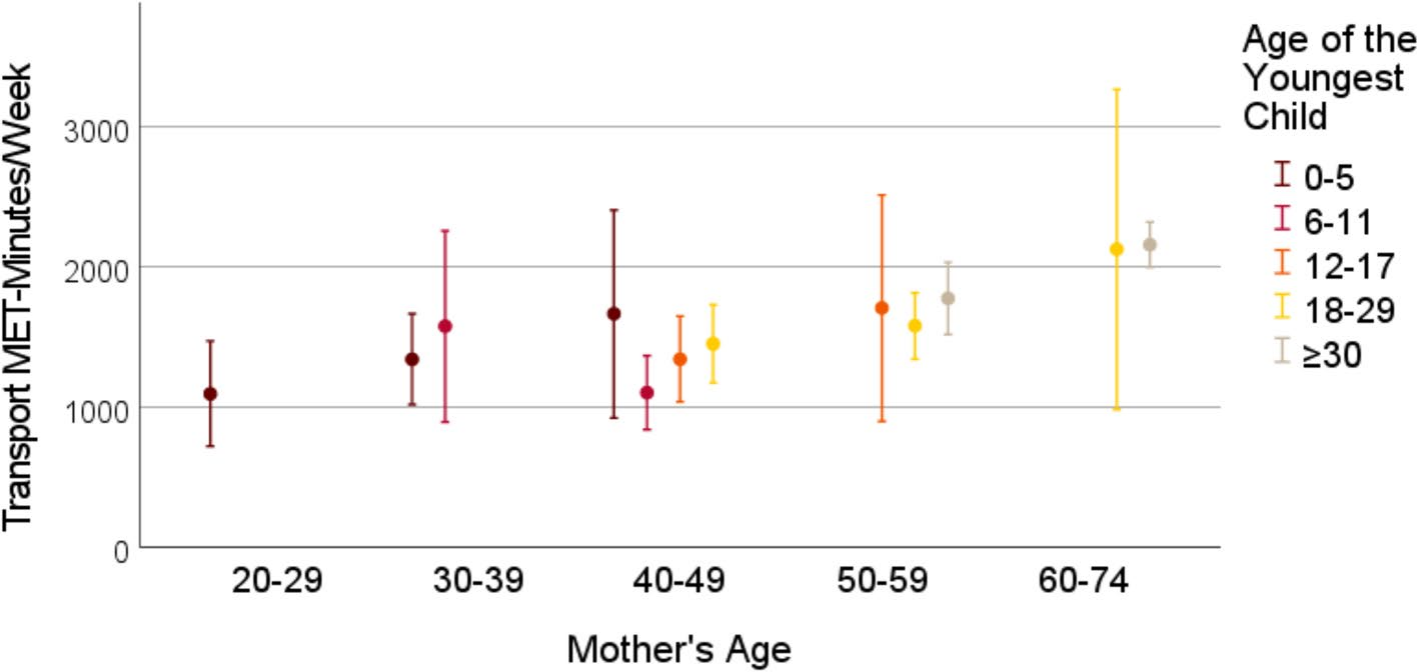
Transport-related MET-minutes per week for subgroups with N > 30 mothers

**Fig. 3:**
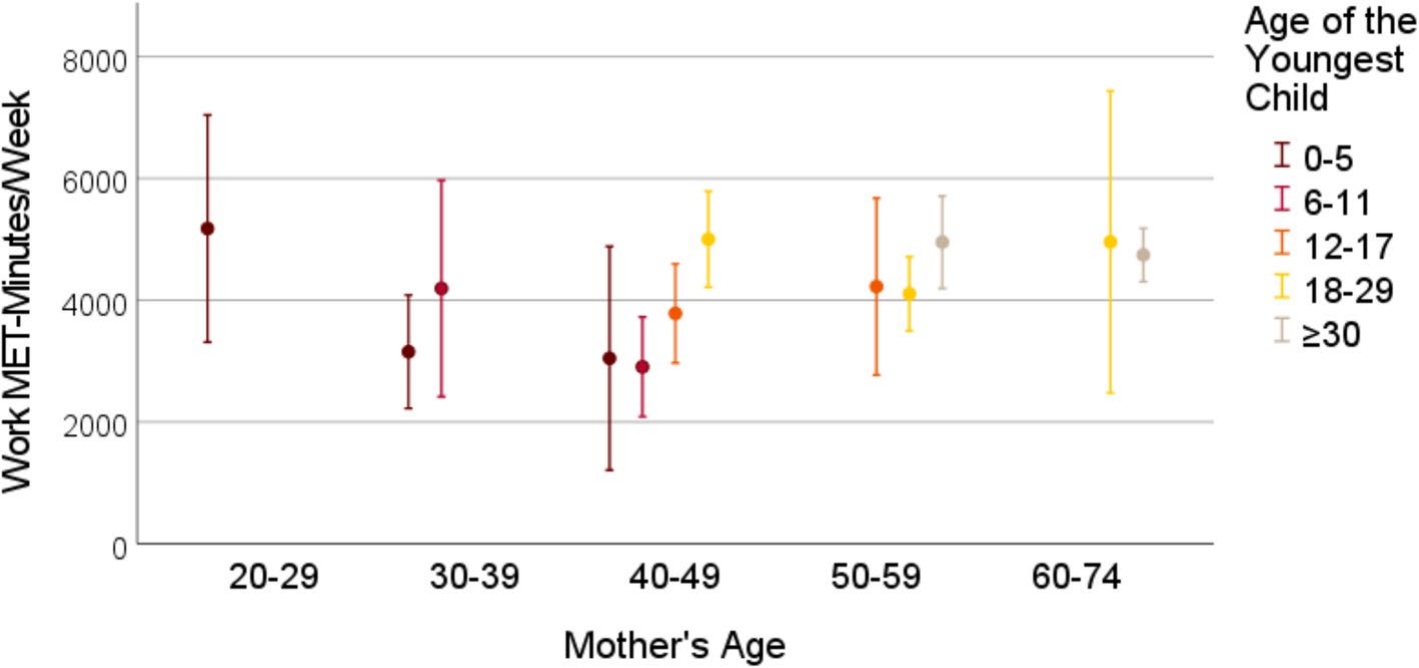
Work-related time MET-minutes per week for subgroups with N > 30 mothers

**Fig. 4:**
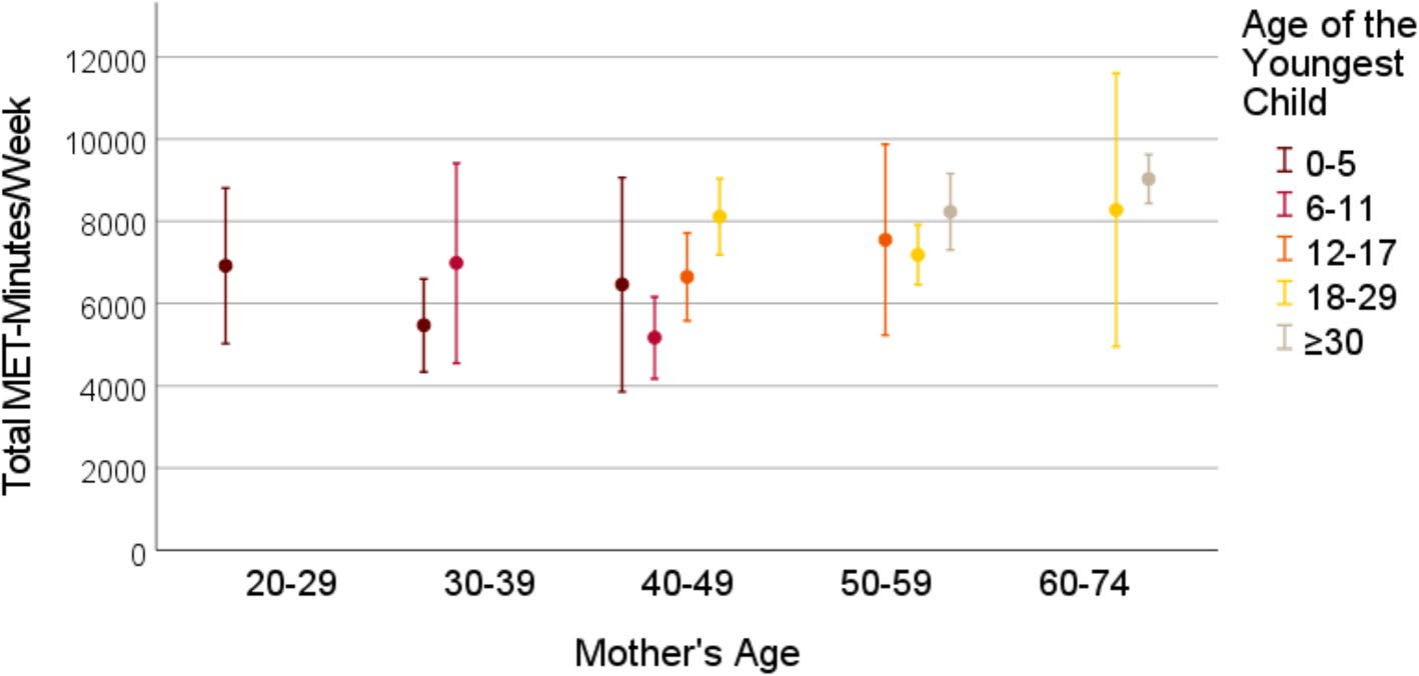
Total MET-minutes per week for subgroups with N > 30 mothers

Figures 1, 2, 3, 4 show the mean MET-minutes per week with 95% confidence interval for each of the three physical activity domains and the total physical activity, stratified by mother`s age and by the age of the youngest child. Mothers at very young as well as at advanced ages had a limited variability in their youngest child’s age. Mothers between the ages 22 and 29 years reached the cutoff group size *N* = 30 only in the youngest child age group from 0–5 years and for the oldest mothers (aged 60–72) the youngest child was either 18–29 or ≥ 30 years old. In middle-aged mothers between 40 and 49 years, four youngest child age groups were at adequate size: 0–5, 6–11, 12–17 and 18–29 years. With respect to the graphs (Figs. 1, 2, 3, 4), the mean MET-minutes of mothers increased with the age of the youngest child in each physical activity category. At the same time, older mother with children in the same age category had lower physical activity. The pattern was similar for all domains of the physical activity, but MET-minutes for work related activity were substantially higher (3 to 4 times higher) than in leisure or transport domains.

The results of the linear regression analyses are shown unadjusted (Table 3) and adjusted (Table 4). The adjustment for mothers’ age had the strongest effect (see supplemental file, additional Table 1).

**Table 3:** The association between the youngest child’s age and mothers’ physical activity by activity domain

	**Leisure Time MET-minutes/Week**	**Transport MET-minutes/Week**	**Work MET-minutes/Week** (including housework)	**Total MET-minutes/Week**
	β (95%CI)	β (95%CI)	β (95%CI)	β (95%CI)
** Child’s Age**				
0–5	-437.2 (-851.7; -22.8)	-359.6 (-790.8; 71.5)	-1809.0 (-2983.7; -634.4)	-2500.6 (-3970.9; -1030.4)
6–11	-330.1 (-697.0; 36.8)	-502.2 (-884.2; -120.3)	-1694.6 (-2739.2; -650.1)	-2502.9 (-3795.8; -1210.1)
12–17	-14.5 (-356.1; 327.1)	-351.4 (-705.1; 2.2)	-887.6 (-1854.4; 79.2)	-1212.8 (-2413.9; -11.7)
18–29	4.3 (-281.6; 290.3)	-256.7 (-555.2; 41.8)	-460.9 (-1275.9; 354.1)	-650.2 (-1660.9; 360.5)
≥ 30	*Ref*			
R^2^	0.003	0.003	0.006	0.008

Beta values with 95% CI indicated for mothers between the ages 30 and 59 years. Unadjusted results

*CI* Confidence interval

*MET* Metabolic equivalent

**Table 4:** The association between the youngest child’s age and mothers’ physical activity by activity domain

	Leisure Time MET-minutes/Week*	Transport MET-minutes/Week*	Work MET-minutes/Week* (including housework)	Total MET-minutes/Week*
	β (95%CI)	β (95%CI)	β (95%CI)	β (95%CI)
** Child’s Age**				
0–5	-493.6 (-1133.2; 146.0)	-132.4 (-799.4; 534.6)	-2108.9 (-3863.5; -354.3)	-2719.8 (-4923.2; -516.3)
6–11	-531.8 (-1029.4; 34.1)	-348.8 (-867.8; 170.3)	-1770.8 (-3142.2; -399.4)	-2604.6 (-4316.8; -892.4)
12–17	-234.9 (-669.8; 199.9)	-116.8 (-569.1; 335.5)	-903.1 (-2098.2; 291.9)	-1174.3 (-2670.3; 321.6)
18–29	-139.5 (-461.5; 182.6)	-148.5 (-485.1; 188.2)	-584.6 (-1472.9; 303.7)	-784.9 (-1894.5; 324.8)
≥ 30	*Ref*			
** Mother’s Age**				
30–39	-109.3 (-667.6; 448.9)	-76.4 (-659.2; 506.4)	1328.2 (-209.2; 2865.5)	1214.2 (-708.6; 3136.9)
40–49	188.4 (-122.3; 499.2)	-243.9 (-568.8; 81.1)	382.5 (-475.3; 1240.3)	199.2 (-874.0; 1272.5)
50–59	Ref			
R^2^	0.006	0.009	0.039	0.032

Beta values with 95% CI indicated for mothers between the ages 30 and 59 years. Adjusted for mothers’ age, education years, partner status and self-rated health

*CI* confidence interval

*MET* metabolic equivalent

^*^adjusted for mothers’ age, education years, partner status and self-rated health

Compared to the total mean in the sample, the difference between mothers with the children 0–5 years old and those with children > 30 years amounted to an approximately 1/threefold lower physical activity. At the population level, this is partly compensated by the contrary association of physical activity with maternal age, because mothers with small children are most often substantially younger. The exclusion of participants with 0 MET-minutes from the linear regression analyses did not lead to substantial changes within these findings (additional Table 2 and 3).

## Discussion

The present study was conducted to assess the association between the youngest child’s age and mothers’ self-reported physical activity. Our results indicate that women with younger children did not report lower levels of physical activity in leisure time and transport settings. Work-related physical activity differed by the age of the youngest child, with lower self-reported physical activity among mothers of children between 0 and 12 years old when compared to mothers of children at age 30 and older. Another finding was that the self-reported levels of mothers’ physical activity tended to be lower in older mothers, if their youngest child was in the same age group.

### Leisure time physical activity

Previous research compared childless women with mothers of children in different ages. Carson et al. reported greater relationship between motherhood and self-reported leisure time physical activity for mothers with 0–5 year old children than among mothers of older children [11]. Two studies suggested that objectively measured moderate to vigorous physical activity was lower in mothers of children aged under 6 years compared to non-mothers, while the association in mothers of older children was weaker [12, 13]. A recent study focusing on the impact of the child’s age on maternal physical activity found that mothers with at least one child under 5 years engaged in less moderate to vigorous physical activity than mothers of older (school-aged in the UK) children [14]. A possible explanation is that decreased leisure time physical activity among mothers of young children resulted from the fact that those mothers experienced greater time constraints than mothers of older children [14]. Lack of time was found to be an important barrier to physical activity among mothers of young children [39–41], which may change during the adolescence of their children [42]. Difficulties arise, however, in comparing these studies with our results due to the lack of information in which activity domains mothers were physically active, especially in the examinations using accelerometer data [12–14]. The previously reported lower levels of physical activity among mothers of young children are not in line with our results that did not indicate differences in mothers’ leisure time physical activity with the youngest child’s age. This may partly be explained by small sample size in some age groups in our analyses, particularly in the group youngest child’s age 0–5 years, among whose physical activity of mothers differed the most in previous research [11–14].

### Transport-related physical activity

To our knowledge, there is no previous research on transport-related physical activity comparing mothers with children in different ages. A current systematic review on physical activity in transport was inconclusive about whether it is impacted by having children in general [43]. Our results suggest that mothers of younger children reported lower levels of transport-related physical activity when compared to mothers of children aged 30 years and older, but confidence interval of the beta value was broad and always included the zero effect. Previous studies did not report differences in transport-related physical activity in relation to parenthood [18, 44–47]. A possible explanation for this might be that transport-related physical activity is determined in large part by environmental and personal factors, including street lighting, public transport frequency and distance of travel [43]. Studies should further investigate whether having children influences transport-related physical activity levels and, how family constellations interact with those environmental factors.

### Work-related physical activity

In our study, work-related physical activity comprised physical activity in domestic tasks, like housework and childcare activities, and physical activity undertaken as part of employment. Our results suggested that mothers with younger children (age 0–12 years) reported lower work-related physical activity levels when compared to mothers of older children. This may be partly explained by the fact that parents of young children, especially mothers, are less likely to be employed [48] or having a full time employment, and therefore have fewer opportunity to engage in occupational physical activity. In previous research, mothers’ working hours were found to be closely linked with the child’s age, particularly when having younger children [49, 50], which may possibly explain the fewest work-related MET-minutes among mothers of the youngest children. With children over the age of 12, some mothers may have increased their working hours, and consequently, the time spent in occupational active behaviors [51]. However, to our knowledge, this is the first study that examined the association between child’s age and mothers’ physical activity in work settings and several questions remain unanswered at present.

### Total physical activity

The results of this study did show that all-domain physical activity of mothers differed with the age of the youngest child. While investigating the physical activity domains separately, the differences with the youngest child’s age were several times higher with regard to mothers’ work-related physical activity than in leisure time or transport physical activity.

### Differences of maternal physical activity with mothers’ age

Among mothers with a youngest child in the same age group, the mothers’ age seemed to be associated with the self-reported maternal physical activity. Older mothers reported deceased time and intensity in physical activity when compared to younger mothers whose youngest child was at same age. This is the first study that reported this finding. This could be simply an effect of ageing, but also there could be differences between cohorts. Older generations were found to engage in less physical activity than recent generations [52] and previous research reported the tendency to maintain active behaviors over the life course [53].

### Strengths and limitations

This was the first study to assess the association between the child’s age and mothers’ physical activity pattern in a large, representative sample of the German population. The analyses offered important insights into maternal domain-specific physical activity.

However, there is a potential bias from the fact that all data was self-reported. Particularly the information on physical activity is susceptible to desirability bias, which may have resulted in overestimation [54]. Data on physical activity comparing assessment tools for physical activity in the German National Cohort showed notable higher physical activity levels in self-reported variables like the GPAQ data when compared to objectively measured physical activity [29]. In particular mothers’ work-related activity levels were relatively high, which may be explained by the fact that the participants were asked to include non-paid work-related activities, such as household and childcare activities. Further, the MET values are reported for those participants who indicated to be active at the particular physical activity domain, excluding those with no reported activity in the specific domain. Due to relatively high proportion of participants that reported not to have any activity, the distribution of the MET values was rather asymmetrical. Another limitation of the GPAQ is that the participants were asked to report information on physical activity, that was absolved during an interval of at least 10 min. Short-time activities (< 10 min) are not represented in the analyses, which may have led to a relatively high proportion of participants who indicated not to be active within each physical activity domain. It is possible that the frequency of these unaccounted intervals (< 10 min) depended on age of the child. Furthermore, physical activity in each domain may not lead to better health to the same extent, and adverse health effects of work-related physical activity on health outcomes are under discussion [55, 56]. In addition, data was restricted to the study center of GNC in Halle (Saale) in the former eastern part of Germany. The distribution of demographic factors differs between the regions in Germany, especially in the case of family arrangements, which may limit generalizability of findings [34, 57]. Another source of uncertainty is that a live birth defined being a mother. The current family constellation and living arrangement at examination time remain unknown. No information was collected on whether the mother was the primary caregiver for the child. Furthermore, additional (possibly younger) stepchildren or adoptive children were not considered. Some women with non-biological children that identified themselves as mothers were possibly excluded from our analyses. Due to the growing significance of such family constellations, future research should explicitly investigate possible associations between having stepchildren or adoptive children and maternal physical activity [58]. Lastly, even though we performed the analyses based on the youngest child’s age, 60,6% of the mothers had two or more biological children. The additional child(ren) may have influenced mothers’ engagement in domain-specific physical activity [14].

There is room for further progress in determining mothers’ physical activity trajectories over their children’s life courses, primarily by applying longitudinal study designs and using both, self-reported and objective measurements for physical activity. Moreover, the results outline the importance of analyzing the domains of physical activity separately in research among mothers.

## Conclusions

The current study confirmed the differences in mothers` physical activity by age of the youngest child, with mothers of youngest children having much lower levels of activity. While there were no clear differences for activity in leisure time and transport settings, the differences in work related activity dominated the results. Mothers’ self-reported physical activity was lower with increased mothers’ age when compared to younger mothers whose youngest child was in the same age group. This could be partly also a cohort effect, with older cohorts having lower levels of physical activity.

### Supplementary Information

Supplementary Material 1. Additional Table 1. The association between the youngest child’s age and mothers’ physical activity by activity domain.

Supplementary Material 2. Additional Table 2. The association between the youngest child’s age and mothers’ physical activity by activity domain.

Supplementary Material 3. Additional Table 3. The association between the youngest child’s age and mothers’ physical activity by activity domain.

## Data Availability

The datasets analyzed during the current study are not publicly available because data of the German National Cohort (NAKO) study are non-public. Data are available via the NAKO Gesundheitsstudie (contact via www.nako.de) on reasonable request.

## Abbreviations

WHO: World health organization
GNC: German national cohort
GPAQ: Global physical activity questionnaire
MET: Metabolic equivalent
ICSED: Standard classification of education
SD: Standard deviation
OR: Odds ratio
CI: Confidence interval
PA: Physical activity
