# Supplementary material for: The association between the child’s age and mothers’ physical activity: results from the population-based German National Cohort study
Source: BMC Public Health. 2024 Jun 13;24:1584. doi: 10.1186/s12889-024-19055-y (PMC11170828; doi:10.1186/s12889-024-19055-y)
Supplement: Supplementary file 1 — Supplementary Material 1. Additional Table 1. The association between the youngest child’s age and mothers’ physical activity by activity domain. [file 12889_2024_19055_MOESM1_ESM.pdf]

	Leisure Time MET-minutes/Week*	Transport MET-minutes/Week*	Work MET-minutes/Week* (including housework)	Total MET-minutes/Week*
	β (95%CI)	β (95%CI)	β (95%CI)	β (95%CI)
**Child's Age**				
0-5	*-362.3 (-1181.0; 456.5)*	*-713.8 (-1633.5; 205.8)*	*-6499.0 (-9702.8; -3295.2)*	*-4092.4 (-6375.9; -1808.9)*
6-11	-604.3 (-1225.4; 16.9)	-562.0 (-1301.8; 177.8)	-4611.9 (-7174.8; -2049.0)	-3449.5 (-5230.4; -1668.5)
12-17	-250.1 (-797.4; 297.1)	-403.7 (-1039.0; 231.5)	-3123.5 (-5229.5; -1017.5)	-1921.9 (-3480.7; -363.0)
18-29	-95.7 (-499.3; 308.0)	-246.5 (-713.8; 220.8)	-1946.7 (-3530.8; -362.6)	-884.6 (-2032.3; 263.1)
≥30	*Ref.*			
**Mother's Age**				
30-39	-152.2 (-870.2; 566.2)	146.2 (-676.1; 968.4)	3645.6 (816.7; 6474.5)	1957.8 (-62.6; 3978.1)
40-49	247.9 (-145.2; 641.1)	-2.0 (-467.5; 463.5)	1903.4 (384.2; 3422.6)	748.2 (-375.9; 1872.2)
50-59	*Ref.*			
R^2	*0.005*	*0.003*	*0.016*	*0.007*

*adjusted for mothers' age

CI: confidence interval
MET: metabolic equivalent
