# Supplementary material for: The association between the child’s age and mothers’ physical activity: results from the population-based German National Cohort study
Source: BMC Public Health. 2024 Jun 13;24:1584. doi: 10.1186/s12889-024-19055-y (PMC11170828; doi:10.1186/s12889-024-19055-y)
Supplement: Supplementary file 2 — Supplementary Material 2. Additional Table 2. The association between the youngest child’s age and mothers’ physical activity by activity domain. [file 12889_2024_19055_MOESM2_ESM.pdf]

	Leisure Time MET-minutes/Week	Transport MET-minutes/Week	Work MET-minutes/Week (including housework)	Total MET-minutes/Week
	β (95%CI)	β (95%CI)	β (95%CI)	β (95%CI)
**Child's Age**				
0-5	*-423.4 (-985.3; 138.4)*	*-603.4 (-1217.0; 10.3)*	*-3299.9 (-5488.5; -1111.3)*	-2449.7 (-4010.1; -889.3)
6-11	-470.2 (-947.1; 6.8)	-524.3 (-1093.3; 44.8)	-2255.3 (-4254.0; -256.6)	-2422.9 (-3794.0; -1050.9)
12-17	-68.2 (-510.7; 374.4)	-395.2 (-911.9; 121.5)	-1567.8 (-3330.9; 195.3)	-1245.1 (-2506.7; 16.5)
18-29	1.8 (-370.8; 374.5)	-247.3 (-680.6; 186.1)	-1178.3 (-2642.7; 286.1)	-593.3 (-1655.5; 468.9)
≥30	*Ref.*			
R^2	*0.004*	*0.003*	*0.009*	*0.008*

CI: confidence interval
MET: metabolic equivalent
