# Supplementary material for: The association between the child’s age and mothers’ physical activity: results from the population-based German National Cohort study
Source: BMC Public Health. 2024 Jun 13;24:1584. doi: 10.1186/s12889-024-19055-y (PMC11170828; doi:10.1186/s12889-024-19055-y)
Supplement: Supplementary file 3 — Supplementary Material 3. Additional Table 3. The association between the youngest child’s age and mothers’ physical activity by activity domain. [file 12889_2024_19055_MOESM3_ESM.pdf]

	Leisure Time MET-minutes/Week*	Transport MET-minutes/Week*	Work MET-minutes/Week* (including housework)	Total MET-minutes/Week*
	β (95%CI)	β (95%CI)	β (95%CI)	β (95%CI)
**Child's Age**				
0-5	-277.6 (-1131.5; 576.3)	-337.0 (-1311.3; 637.3)	-4031.1 (-7296.9; -765.3)	-2762.5 (-5097.7; -427.3)
6-11	-603.2 (-1251.7; 45.3)	-442.1 (-1223.4; 339.3)	-3089.0 (-5660.3; -517.8)	-2621.2 (-4433.7; -808.6)
12-17	-229.5 (-799.2; 340.2)	-170.4 (-836.7; 495.9)	-2301.8 (-4402.9; -200.6)	-1294.9 (-2873.0; 283.2)
18-29	-128.2 (-546.4; 289.9)	-201.4 (-688.1; 285.4)	-1887.4 (-3461.2; -313.6)	-798.9 (-1958.6; 360.8)
≥30	*Ref.*			
**Mother's Age**				
30-39	-122.2 (-861.5; 617.2)	45.3 (-808.2; 898.7)	1996.3 (-825.8; 4818.4)	1625.4 (-410.7; 3661.5)
40-49	278.3 (-129.3; 686.0)	-68.8 (-552.4; 414.8)	1372.7 (-142.7; 2888.1)	525.4 (-608.9; 1659.7)
50-59	*Ref.*			
R^2	*0.009*	*0.014*	*0.079*	*0.039*

*adjusted for mothers' age, education years, partner status and self-rated health

CI: confidence interval
MET: metabolic equivalent
